# The Square Curve Paradigm for Research in Alternative, Complementary, and Holistic Medicine: A Cost-Effective, Easy, and Scientifically Valid Design for Evidence-Based Medicine and Quality Improvement

**DOI:** 10.1100/tsw.2003.97

**Published:** 2003-11-13

**Authors:** Soren Ventegodt, Niels JØrgen Andersen, Joav Merrick

**Affiliations:** The Quality of Life Research Center, Teglgårdstræde 4-8, DK-1452 Copenhagen K, Denmark; Norwegian School of Management, Sandvika, Norway; National Institute of Child Health and Human Development, Office of the Medical Director, Division for Mental Retardation, Ministry of Social Affairs, Jerusalem, Israel

**Keywords:** human development, holistic medicine, public health, consciousness, evidence-based medicine, research methodology, quality of life, Denmark, Norway, Israel

## Abstract

In this paper we present a new research paradigm for alternative, complementary, and holistic medicine — a low-cost, effective, and scientifically valid design for evidence-based medicine. Our aim is to find the simplest, cheapest, and most practical way to collect data of sufficient quality and validity to determine: (1) which kinds of treatment give a clinically relevant improvement to quality of life, health, and/or functionality; (2) which groups of patients can be aided by alternative, complementary, or holistic medicine; and (3) which therapists have the competence to achieve the clinically relevant improvements. Our solution to the problem is that a positive change in quality of life must be immediate to be taken as caused by an intervention. We define “immediate” as within 1 month of the intervention. If we can demonstrate a positive result with a group of chronic patients (20 or more patients who have had their disease or state of suffering for 1 year or more), who can be significantly helped within 1 month, and the situation is still improved 1 year after, we find it scientifically evidenced that this cure or intervention has helped the patients. We call this characteristic curve a “square curve”. If a global, generic, quality-of-life questionnaire like QOL5 or, even better, a QOL-Health-Ability questionnaire (a quality-of-life questionnaire combined with a self-evaluated health and ability to function questionnaire) is administered to the patients before and after the intervention, it is possible to document the effect of an intervention to a cost of only a few thousand Euros/USD. A general acceptance of this new research design will solve the problem that there is not enough money in alternative, complementary, and holistic medicine to pay the normal cost of a biomedical Cochrane study. As financial problems must not hinder the vital research in nonbiomedical medicine, we ask the scientific community to accept this new research standard.

## INTRODUCTION

Today every second person in Scandinavia is chronically ill, if you include minor diseases like allergies, eczema, lower back pain, or migraine, and 94% of the population in Denmark has one or another symptom of ill health[1]. The National Health Service, supplied free or almost free of charge in Scandinavia, is being supplemented to an increasing degree by alternative, complementary, and holistic medical services paid for by the individual user. There are numerous anecdotes of patients with cancer, arteriosclerosis, tinnitus, schizophrenia, and other serious or incapacitating diseases being helped or even cured by these treatments[2], but at the same time, there is no evidence that explains the spontaneous remissions from schizophrenia (ten international studies with 25–35 year follow-up show 46–64 % complete or near complete recovery) or cancer and not much connects these remissions to any kind of treatment.

One reason for the scarcity of scientific evidence could be that the extensive use of alternative and holistic therapy — 800,000 patients use it now in Denmark or about 15% of the population[3] — is actually not linked to the improvement of health of the patients. Another and much more likely reason for the scarcity of documented success could be that the economic interest in alternative and holistic medicine so far has been much too small to finance the necessary research. It is, however, of utmost importance that nonbiomedical medicine is carefully examined for possible positive effects on a variety of different diseases and human states of suffering, since one must suspect that some of the alternative and holistic medicine provided by the most competent of the therapists do actually help at least some patient groups.

Let us therefore think about ways to solve that problem. If the problem is getting the money for research, one way is to raise more money, but that is obviously the hard road. Another way is to make the research cheaper. It seems that this is possible if we can agree on some rules. As we definitely do not want to accept a method with less evidence, plausibility, certainty, and validity, we have to design a new method giving all these fine qualities of science, but at a cost of about 1% of the normal budget for research.

## THE THEORETICAL ADVANTAGE OF ALTERNATIVE AND HOLISTIC MEDICINE

Alternative treatment is normally defined as treatment that is not provided by established health services and providers. Since many physicians have started to use alternative methods in their practices, most commonly a simple form of acupuncture, it is necessary to define alternative treatment as a treatment that, in contrast to biomedicine, builds openly on the self-healing resources and potentials of the patient him/herself. This is, of course, true for any treatment from the time of Hippocrates until today, but much modern medicine has forgotten this. In alternative and holistic medicine, these hidden resources are considered a vital part of the human whole. In most kinds of alternative medicine these self-healing resources are triggered into use by helping the patients to change attitudes, resulting in general improvement of the human global quality of life, health, and ability to function. That is why we use the expression consciousness-oriented medicine about the “alternative and holistic medicine” in this paper and also why we imagine that, in spite of the obvious lack of evidence, the best alternative and holistic medicine might actually have the power to help.

The empowerment of the patient though personal development mobilizes these hidden resources and this “medicine” has many advantages. It is affordable for all patients, it is cheap for the society, a benefit to the workplace, it does not deprive the patient of the responsibility for his or her own existence, it is “organic”, takes care of the ecosystem, it does not pollute, it has almost no side effects, and finally it might give the patient a permanent benefit if successful. If the person who takes his hidden resources into use does this so well as to improve the global quality of life, this person might even get less sick in the future, and be of greater value to his or her surrounding world. So we can say that this consciousness-oriented medicine might also be preventive and societally constructive.

## CAN WE HAVE GOOD MEDICAL SCIENCE WHEN WE LACK THEORY AS WELL AS RATIONAL METHODOLOGY?

Research can be a value-enhancing activity. This happens when research produces data of necessary certainty and quality, and the data are produced in relation to the relevant endpoints and the basic intention for the research. If, on a scientific basis, one wishes to improve the subjective experience of health, one must measure psychometrically to retrieve the data necessary to evaluate if the experience of one’s own health has become sufficiently improved, in relation to the intention. That is why we must keep track of both intention and endpoints in research, and secure that these are always aligned with each other.

One serious difficulty with research in consciousness-based medicine is that alternative and holistic treatments seldom live up to the general demands of scientific theory and method. Therefore the research must, if at all possible, be arranged so it can be used on all forms of treatment, regardless of the theory and method underlying this treatment.

When research shows that an alternative or holistic treatment gives the patients a significant improvement, there is apparently something to gain from this treatment. This documentation is in itself of great value. Of course this is not the end of the research, but only the beginning. New research projects must now be established to clarify the theory and method. Only when the method of treatment is understood, described rationally, and linked to sound scientific theory, can we make a valid scientific contribution to the treasure of medical knowledge. A scientifically trained doctor will never use meaningless rituals or give substances he considers useless to the patient, no matter how well it works for an alternative therapist. Without scientific understanding, an alternative or holistic cure will never be used by the medical community. But without examining the effects of the new or alternative cures and interventions, medicine will never be able to develop in a positive direction.

Below, we suggest a simple and scientifically trustworthy research design, which can be accomplished within the economical limits of a small public or private research center or corresponding organization. It has been developed to study the effects of alternative and holistic therapies at the “Frisklivssenteret” (the “Healthy Life Centre”) in Porsgrunn, Norway, in collaboration with the independent nonprofit organization “The Scandinavian Foundation for Holistic Medicine”.

**TABLE 1**
**The Points to be Included in Surveys with Adults**

**Quality of life**
Self-evaluated, global quality of life
**Health**
Self-evaluated physical health
Self-evaluated psychic health
**Ability to function**
Self-evaluated, global ability to function, assessed in subdimensions, corresponding to life's three dimensions of leisure time, work, and family:

- Self-evaluated social functionality
- Self-evaluated working ability
- Self-evaluated functionality concerning love
- Self-evaluated functionality concerning sexuality

**TABLE 2**
**The Points to be Included in Surveys with Children**

**Quality of life**
Self-evaluated, global quality of life
Parent-evaluated, global quality of life
**Health**
Parent-evaluated physical health
Parent-evaluated mental health
**Ability to function**
Parent-evaluated global ability to function, evaluated by the following subdimensions:

- Parent-evaluated functionality in the family
- Parent-evaluated functionality at school/institution
- Parent-evaluated social functionality with the same and opposite sex

## SCIENTIFIC DEMANDS FOR EVIDENCE-BASED ALTERNATIVE AND HOLISTIC MEDICINE

We make the following demands on the quality and validity of the collected results:

1. The results are qualitatively meaningful and the applied endpoints correspond closely to the intention behind all sound alternative and holistic treatment, namely the general improvement of quality of life, health, and functionality. It is these dimensions that must be measured in the study (see Tables 1 and 2).
2. The quantitative results are valid[4] and statistically significant at a $p < 0.05$ level (95% probability that the proven effect is true).
3. The results are visibly and directly observed as an immediate consequence of a treatment. By immediate we mean within 1 month from the start of the intervention. A survey before and after the intervention must show a significant and clinically relevant improvement of the patient’s condition regarding self-evaluated quality of life, health, and/or functionality. Is this case, a control group is not needed.

4. The questionnaire(s) must be appropriate and validated. They must have the correct amount of questions necessary to document a clinically and statistically significant improvement of the relevant dimensions. We accept an average improvement of one-half point on a five-point Likert scale as adequate to call a treatment good, but an improvement of one-quarter of a point is of clinical relevance.
5. There must be at least 20 patients in the group receiving the treatment, and the patients must be sufficiently well characterized to allow falsification of the formulated hypothesis: Therapist (x) can, with method (y), help patients with diagnosis (z), in the age interval (p), the degree of motivation (q), the resources (r), etc. improve their quality of life/physical health/psychological health/ability to function.

A simple way to ensure valid results is to demand that the results are significant and produced immediately (within 1 month). This intervention can naturally be repeated a desired number of times to increase or secure a result of treatment. In case of repeated treatments, ratings of endpoints must show a positive trend on a run-chart[5]. The rating is repeated again after an appropriate length of time to see the long-term effect of the treatment, at least 1 year after the initial treatment.

The ideal form of the curve that can document significant effects from alternative and holistic medicine is in fact "square" (see Fig. 1); an even better curve is an upward slope as quality of life, health, and ability to function continues to improve after the intervention[6]. The patient baseline level regarding quality of life and/or health and/or functionality is lifted, within 1 month after the initiation of the intervention, to a new, significantly higher and stable level of quality of life and/or health and/or functionality. Since these three factors are closely related statistically[1,7], it is expected that the patients will receive a similar increase in both quality of life, health, and functionality, but an improvement in one of these three dimensions alone is acceptable for a treatment to be of value.

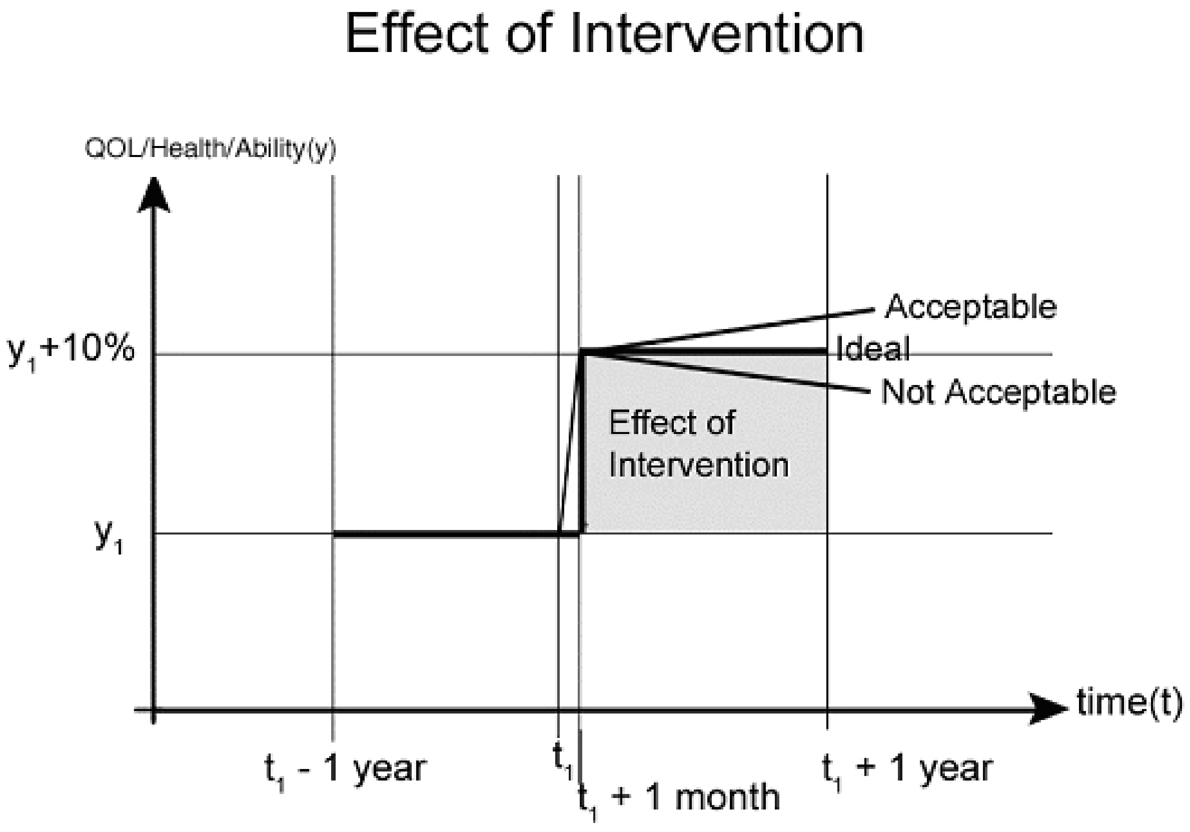

**FIGURE 1**. The “square” curve. The ideal curve that documents scientifically valid and clinically relevant effect of alternative and holistic medicine is squared, since the group, on average, is on one level before and a higher level after the treatment. The treatment intervention must be of limited duration. Endpoints are QOL, Health and/or Ability of Functioning. The area under the square curve (grey) is the documented gain of the patients.

For the square curve to appear time must be measured at *T1 minus one year* (sufficiently long time before the intervention), at *T1* (immediately before the intervention), at *T1 plus one month* (immediately after the intervention), and at *T1 plus one year* (sufficiently long time after the intervention). If the patients are chronically ill and the situation is stabile, the measurement *T1 minus one year* is not necessary.

Please note that if the effect is lost through time, which is when the difference between Y(*T1 plus one year) and* Y(*T1 plus one month)* is negative and larger that the insecurity of the measurement, the effect is not documented (the curve marked “not acceptable”).

In Fig. 1, the x-axis represents time and the y-axis represents condition of quality of life/health/functionality. The leap up has to be clinically significant and must be as well defined as possible. It must come as a consequence of our intervention so there can be no doubt that it is caused by it. Whether the intervention is made one patient at a time and summed up to the collective curve, or all patients are treated at once, has no significance for the scientific validity of the documentation. The state of quality of life, health, and/or ability to function must have been at the baseline level for at least 1 year. The improvement must be found unchanged 1 year after the intervention. If this is the case, there is no need for a control group.

## Comments to Point One Above

Endpoints must always correspond to the intention of the treatment. In the case of alternative and holistic medicine, the intention is a general improvement of quality of life, health, and functionality. It is these dimensions that are to be measured, in a way that will make comparison possible. Since we are facing a common intention, we can measure all enterprises with the same endpoints, which make the research very rational and thereby economically reasonable. This is how it must be when hundreds of kinds of treatment, hundred different groups of patients, and hundreds of different therapists are to be submitted to evidence-based medical research.

## Comments to Point Two Above

Results can be documented quantitatively or qualitatively. Qualitative documentations that are often based on the patient’s own statements are difficult and demand large resources to compare and are not very reliable. A patient may feel that he/she has received good help even though quality of life, health, and functionality have not been improved, i.e., that a patient is aided in resignation, or that symptoms are suppressed without the existing suffering being treated. It is not possible to statistically analyze qualitative data. Therefore, to be certain of documenting an improvement, we have to demand numbers that can show the improvement in a simple way, and fulfill the demands of medical science for statistical analysis, namely a generally accepted probability of 95%. This kind of data can easily be provided by using psychometric tools; namely appropriate and validated questionnaires.

## Comments to Point Three Above

One big difficulty with alternative treatment is that one seldom, in a sensible way, can create a blinded control group. One can create a group that does not receive the treatment that is offered, but since many alternative therapies are accessible on the market in one form or another, one cannot in any way prevent those who are not included in the test group from buying a corresponding service outside the normal program. Since a substantial part of the healing on a long-term basis comes as a consequence of the initiatives and programs people do on their own, one cannot exclude, if an intervention takes place over a certain period of time, that a corresponding healing in a motivated control group is caused by a corresponding treatment. Thereby the control group loses its fundamental idea and validity. One can argue that if the intervention is no better if the control group finds out and fixes themselves, then the treatment given is unnecessary, but this is not a valid statement, as people pay for these services and they are often willing to pay a surprising amount of money for them, often thousands of Euro/USD. To know if they actually help people is therefore an important question.

It is difficult to document progress with alternative treatment when the intervention lasts for a long time. Many alternative systems of treatment let patients go on for years waiting for progress that may come. That is not what we want to offer our patients in modern medical science, and that is why that kind of treatment is not in correspondence with the intention of effective improvement of life quality, health, and functionality. Thus it is important that the treatments given can show rapid and visual results.

The reason why we say that 1 month is “immediate” is that, within 1 month, it is very unlikely that something happens that changes the quality of a patient’s life. On the other hand, it is sufficient time to give at least eight sessions of most therapies. If eight sessions do not create a visible result in at least some of the patients, giving a significant rise in the measured quality of life, health, and ability to function, we take it that the cure is not effective.

## Comments to Point Four Above

At the moment, there are economical limitations restricting the research of alternative and holistic medicine. Therefore documentation must be necessary and adequate, but not more. Since what troubles the patients is always their own experience of life quality, health, and functionality, it is sufficient to examine this to document the effect we wish to give our patients by alternative and holistic medicine. Objective examinations are desirable, but very expensive, and are not necessary at this stage.

Psychometric tools; questionnaires concerning quality of life, health, and functionality that measure exactly what they should and that have the correct amount of questions that are necessary to prove a clinically essential improvement of the relevant dimensions, are therefore the right choice for this research. One works on a five-point Likert scale with a neutral center as in the extremely short, validated QOL1 questionnaire [8]:

How do you assess the quality of your life now?

1. Very high
2. High
3. Neither high nor low
4. Low
5. Very low

The difference in quality of life, health, and functionality required to call a treatment clinically relevant (clinically significant) must be one-fourth of the difference between the point of the scale, and a good intervention must raise the intervention group an average of at least one-half step on the scale. The rationale for this is that people with a global quality of life rating below 3.5 on a five-point symmetrical Likert scale (between “high” and “neither high nor low”, calculated as 60%) often are unable to work while people above 4.0 (“high” calculated as 70%) often are able to work. Being able to work seems to be an important indicator of resources and health, and we want a valid treatment to be able to bring people back to work.

If the patients are on a level of “low” at the beginning, the therapist must lift the group to a level between “low” and “neither high nor low”. This will not make the patient able to work, but the improvement is thought to be of as much value to the individual. It is obvious that an optimal treatment will lift the group four or even six times as much, namely to the level “high” or “very high”. But for a single intervention taking less than 1 month, we are willing to acknowledge the increase of one-half step on a five-point Likert scale as fine and remarkable. An improvement of one step (20%) is acknowledged as excellent.

## Comments to Point Five Above

The desired difference of one-half a step (calculated as 10%[9]) can be statistically significantly documented (with the given one-half steps on the scale as above) with 19 patients, as shown for the question of quality of life (QOL1)[8]. If five questions are used (QOL5[8]), a difference of one-quarter step can easily be detected with 20 patients. Therefore, the number of 20 people is adequate for a statistically significant measuring of the difference, before compared to after the intervention.

Global quality of life and self-assessed health (physical and mental) can be measured with the QOL1 and QOL5 questionnaires[8]. Global ability of functioning must be measured by an appropriate questionnaire (in preparation).

## DISCUSSION

The proposed research design is not without difficulties, as psychometrics is a complex issue, and consciousness in general is poorly understood. A consciousness-based medicine is basically using changes in consciousness, well exemplified by the placebo effect that is such an annoyance in biomedical research. Working directly on the consciousness is of course infinitely more powerful that just tricking it with a pill.

The presented square curve paradigm aims to eliminate the highly esteemed control group of traditional clinical testing. Before judging the scientific value of this paradigm, please consider the fact that the standard procedure in biomedical research using the control group is not without difficulties either. When a company selects drugs that give the patients an internal sensation of receiving a drug, they boost the placebo effect (“active placebo”) and so the “blinded” test is not at all blind, since all patients should have a similar experience of getting an active drug. So the control group in a fair trial should also be “boosted” with an internal clue of receiving a drug. The placebo effect is known to be enhanced dramatically by this internal clue, as this was the reason when physicians administrated strychnine and other poisonous substances to their patients for centuries with absolutely no specific therapeutic effect. So please do not be naive about the validity of even the finest scientific designs.

The best proof for a clinically significant effect of an intervention is that our patients actually improve their health, feel better today, and that they stay this way. This is the case when our patient with problem X gets intervention Y? This is exactly what the square curve paradigm tells us to test for, in a reliable way. A problem with the square curve paradigm is of course that it is insensitive to slow improvements made over a longer time, but this can be made visible using statistical process control methods like run-charts.

## CONCLUSION

If the scientific community can acknowledge an effect on quality of life (QOL), health, and ability to function to be caused by an intervention, if the patients have been in a stable state for a year, if they are raised to a better state within a month, and if they stay in this better state for another year, the effect of alternative and holistic medicine can be evaluated effectively and scientifically with very simple and affordable means. A simple, easily administered, quickly answered, validated, and adequate questionnaire containing less than 100 questions on quality of life, health, and functionality can give the necessary data of sufficient quality for evidence-based alternative and holistic medicine. Ideally the questionnaire(s) contain(s) no more than 20 core questions supplied by the necessary background information on name, age, sex, diagnosis, etc. Any therapist who wishes to be a part of the study can easily administrate the questionnaire to his or her patients. Studies are easy to do and can include any kind of alternative and holistic treatment, any groups of patients, recruited from hospitals, clinics, practicing physicians, homes for the mentally ill, or any other organizations of treatment and care.

Thus, with this new suggested "square curve" research paradigm for consciousness-based medicine, it will be possible, with a minimum of resources, to collect the necessary evidence to answer the three fundamental questions in alternative, complementary, and holistic medicine, namely:

- **Who can be helped?** Which group of patients can benefit from alternative or holistic treatment? We know that an important factor in healing is the patient's own degree of competence, resources, and motivation. Must patients be subdivided into groups according to motivation to understand the process of healing?
- **What helps?** Which kinds of alternative and holistic treatment give, with certainty, a clinically relevant improvement of the patient's quality of life, health, and/or functionality?
- **Who can help?** In the Nordic countries, there is an explosive increase of alternative and holistic therapists, available forms of treatment, and alternative institutes training new therapists, giving diplomas to everybody who pays the fee often more or less regardless of the obtained skills. It is therefore important to find out which therapists are competent, and what one must demand from an alternative or holistic therapists' competence.

Every year, thousands of new alternative and holistic therapists and several new kinds of treatment enter the market, but the population surveys show no improvement of the public health. The most likely explanation is that the methods often are not very efficient, or that the competence of the alternative therapists often is too modest to give the patients clinically relevant improvement.

When the therapist works primarily with the consciousness of the patients, it is very difficult to identify faults and errors in the alternative and holistic treatments. The necessary research in alternative and holistic medicine will show which methods (together with the associated theories) are good tools to support the patient's personal development of quality of life, health, and functionality. It is of great value if the research also can document the level of competence of a therapist giving the treatments.

The suggested research design — where several therapists, treatment systems, and patient groups are included in a time-limited study with the improvement of quality of life, health, and functionality as endpoints — is believed to be scientifically reasonable, financially sound, practical, and does not compromise the patients ethically or otherwise, naturally providing that standard research customs are upheld, i.e., confidentiality regarding collected data, written patient documentation, etc. The square curve paradigm is also a general method for quality improvement of any treatment, which takes place in the timespan of 1 month or less, which is supposed to give a lasting improvement, and which has the purpose of improving quality of life, health, and ability to function.

## ACKNOWLEDGMENTS

We want to thank Michael de Vibe, M.D., for his critique of this paper. This report presented results and ideas from the Danish Quality of Life Population Survey, the planning and execution of which would not have been possible without the aid of Dorte Loldrup Poulsen, Jørgen Hilden, and the late Bengt Zachau-Christiansen. Funds were received from nine Danish Foundations, including The 1991 Pharmacy, the

Goodwill, the JL, E. Danielsens & Wife's, Emmerick Meyer's, the Frimodt-Heineken, the Hede Nielsen Family, Petrus Andersen's, C.P. Frederiksen's and the Wedell-Wedellsborgs Foundations, and IMK Almene Fond. The research was approved by the Copenhagen Ethical Committee under numbers (KF) V 100.1762/90 and (KF) 01-502/93.

## BIOSKETCHES

**Søren Ventegodt, MD,** is the Director of the Quality of Life Research Center in Copenhagen, Denmark. He is also responsible for a Research Clinic for Holistic Medicine in Copenhagen and is a popular speaker throughout Scandinavia. He has published numerous scientific or popular articles and a number of books on holistic medicine, quality of life, and quality of working life. His most important scientific contributions are the comprehensive SEQOL questionnaire, the very short QoL5 questionnaire, the integrated QOL theory, the holistic process theory, the life mission theory, and the Danish Quality of Life Research Survey, 1991–94 in cooperation with the University Hospital of Copenhagen and the late pediatric professor Bengt Zachau-Christiansen. E-mail: ventegodt@livskvalitet.org. Website: www.livskvalitet.org/

**Niels Jørgen Andersen, MSc,** Professor, Department of Innovation and Economic Organization, Norwegian School of Management. This department conducts research and provides teaching in central topics related to innovation, business development, management of global companies, business history, and economic organization. Research activities within the Department are related to four core subjects within the discipline: business history, cooperative organizations, business development and entrepreneurship, and finally studies of industries with a special focus on the electricity industry. He is also the dynamic chairman of the nonprofit organization Stiftelsen Holistisk Medisin Scandinavia, which aims to support the scientific development, research, and documentation of complementary and holistic medicine in Scandinavia. E-mail: niels.j.andersen@bi.no. Website: www.bi.no/users/fgl93013/

**Joav Merrick, MD, DMSc,** is Professor of Child Health and Human Development affiliated with the Zusman Child Development Center and Division of Community Health at the Ben Gurion University, Beer-Sheva, Israel and presently the Medical Director of the Division for Mental Retardation, Ministry of Social Affairs, Jerusalem and the Director of the National Institute of Child Health and Human Development. He has numerous publications in the field of child and human development, rehabilitation, intellectual disability, disability, health, welfare, abuse, advocacy and prevention. Dr. Merrick received the Peter Sabroe Child Award for outstanding work on behalf of Danish Children in 1985 and the International LEGO-Prize ("The Children's Nobel Prize") for an extraordinary contribution towards improvement in child welfare and well being in 1987. E-mail: jmerrick@internet-zahav.net. Website: www.nichd-israel.com
